# The ability of digital breast tomosynthesis to reduce additional examinations in older women

**DOI:** 10.3389/fmed.2023.1276434

**Published:** 2023-11-21

**Authors:** Maha Gharaibeh, Ahmad Abu Alfwares, Eyhab Elobeid, Ruba Khasawneh, Liqa Rousan, Mwaffaq El-Heis, Mooath Al-Jarrah, Ahmed A. Haj Hussein, Maryam Altalhi, Laith Abualigah

**Affiliations:** ^1^Department of Diagnostic and Interventional Radiology, Faculty of Medicine, Jordan University of Science and Technology, Irbid, Jordan; ^2^Department of Radiation Technology, Faculty of Applied Medical Sciences, Jordan University of Science and Technology, Irbid, Jordan; ^3^Department of Emergency, Faculty of Medicine, Omdurman Islamic University, Omdurman, Sudan; ^4^Department of Management Information Systems, College of Business Administration, Taif University, Taif, Saudi Arabia; ^5^Computer Science Department, Al al-Bayt University, Mafraq, Jordan; ^6^Department of Electrical and Computer Engineering, Lebanese American University, Byblos, Lebanon; ^7^Hourani Center for Applied Scientific Research, Al-Ahliyya Amman University, Amman, Jordan; ^8^MEU Research Unit, Middle East University, Amman, Jordan; ^9^College of Engineering, Yuan Ze University, Taoyuan, Taiwan; ^10^School of Computer Sciences, Universiti Sains Malaysia, Pulau Pinang, Malaysia; ^11^School of Engineering and Technology, Sunway University Malaysia, Petaling Jaya, Malaysia; ^12^Applied Science Research Center, Applied Science Private University, Amman, Jordan

**Keywords:** digital breast tomosynthesis (DBT), diagnostic performance, breast density, sensitivity, unnecessary additional examinations

## Abstract

**Aims:**

To assess the diagnostic performance of digital breast tomosynthesis (DBT) in older women across varying breast densities and to compare its effectiveness for cancer detection with 2D mammography and ultrasound (U/S) for different breast density categories. Furthermore, our study aimed to predict the potential reduction in unnecessary additional examinations among older women due to DBT.

**Methods:**

This study encompassed a cohort of 224 older women. Each participant underwent both 2D mammography and digital breast tomosynthesis examinations. Supplementary views were conducted when necessary, including spot compression and magnification, ultrasound, and recommended biopsies. Sensitivity, specificity, positive predictive value (PPV), negative predictive value (NPV), and area under the curve (AUC) were calculated for 2D mammography, DBT, and ultrasound. The impact of DBT on diminishing the need for supplementary imaging procedures was predicted through binary logistic regression.

**Results:**

In dense breast tissue, DBT exhibited notably heightened sensitivity and NPV for lesion detection compared to non-dense breasts (61.9% vs. 49.3%, *p* < 0.001) and (72.9% vs. 67.9%, *p* < 0.001), respectively. However, the AUC value of DBT in dense breasts was lower compared with non-dense breasts (0.425 vs. 0.670). Regarding the ability to detect calcifications, DBT demonstrated significantly improved sensitivity and NPV in dense breasts compared to non-dense breasts (100% vs. 99.2%, *p* < 0.001) and (100% vs. 94.7%, *p* < 0.001), respectively. On the other hand, the AUC value of DBT was slightly lower in dense breasts compared with non-dense (0.682 vs. 0.711). Regarding lesion detection for all cases between imaging examinations, the highest sensitivity was observed in 2D mammography (91.7%, *p* < 0.001), followed by DBT (83.7%, *p* < 0.001), and then ultrasound (60.6%, *p* < 0.001). In dense breasts, sensitivity for lesion detection was highest in 2D mammography (92.9%, *p* < 0.001), followed by ultrasound (76.2%, *p* < 0.001), and the last one was DBT. In non-dense breasts, sensitivities were 91% (*p* < 0.001) for 2D mammography, 50.7% (*p* < 0.001) for ultrasound, and 49.3% (*p* < 0.001) for DBT. In terms of calcification detection, DBT displayed significantly superior sensitivity compared to 2D mammography in both dense and non-dense breasts (100% vs. 91.4%, *p* < 0.001) and (99.2% vs. 78.5%, *p* < 0.001), respectively. However, the logistic regression model did not identify any statistically significant relationship (*p* > 0.05) between DBT and the four dependent variables.

**Conclusion:**

Our findings indicate that among older women, DBT does not significantly decrease the requirement for further medical examinations.

## 1 Introduction

Two-dimensional (2D) digital mammography is widely recognized as the gold standard for breast cancer screening (1). It is the most efficient approach for detecting breast cancer (2, 3). A recent Swedish study examined 549,091 women stated that screening mammography leads to 41% reduction in the risk of breast cancer-related mortality within 10 years (relative risk, 0.59; 95% CI, 0.51–0.68 [*p* < 0.001]) and a 25% decrease in the rate of advanced breast cancers (relative risk, 0.75; 95% CI, 0.66–0.84 [*p* < 0.001]) (4). Despite the benefits of 2D mammography, it has a particular limitation represented by tissue overlap that contributed negatively to its sensitivity and specificity, particularly in dense breasts. The main consequence of overlapping tissue is the obscuring of the target tissue and false negative findings, which partially explain the 15–30% missed breast cancers during standard screening. Moreover, the superimposition of normal tissue in the breast can create a pseudo-lesion, usually called a summation artifact. This artifact can lead to false-positive findings that require further investigations, such as follow-up examinations and biopsies. All these additional procedures have a known effect of creating anxiety and leading to non-attendance for breast screening tests among examined women (5, 6).

It has been stated that the probability of developing breast cancer among women with dense breasts is three to six times greater than those with non-dense breasts (7, 8). In addition, the risk of interval cancers (those detected in the interval between planned screening mammograms) is 13–18 times higher among women with dense tissue compared with fatty breasts (9). There is a strong association between breast cancer and aging, as it is considered the leading cause of cancer-related mortality among older women aged 65. Around 41% of all breast cancer cases and 57% of all breast cancer-related deaths occur among older women (10). Moreover, a clinical trial study stated that the risk of breast cancer mortality was 25% higher for women aged between 65 and 74 and 63% more significant for women aged 75 years and more when compared with women under the age of 65 years (11).

**Figure 1:**
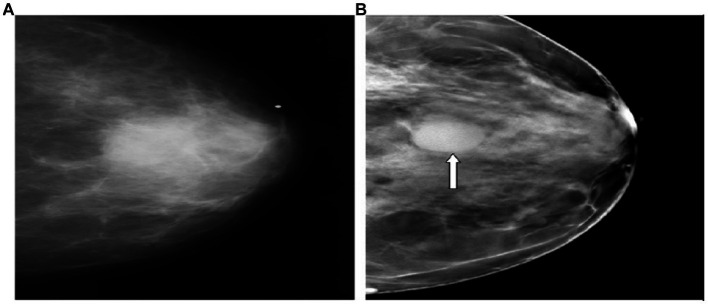
Superiority of DBT over 2D mammography in cancer detection. **(A,B)** Represent the CC view. **(A)** (2D mammogram), the cancer is vaguely apparent. **(B)** The tomosynthesis image depicts a circumscribed mass (arrow) (16).

**Figure 2:**
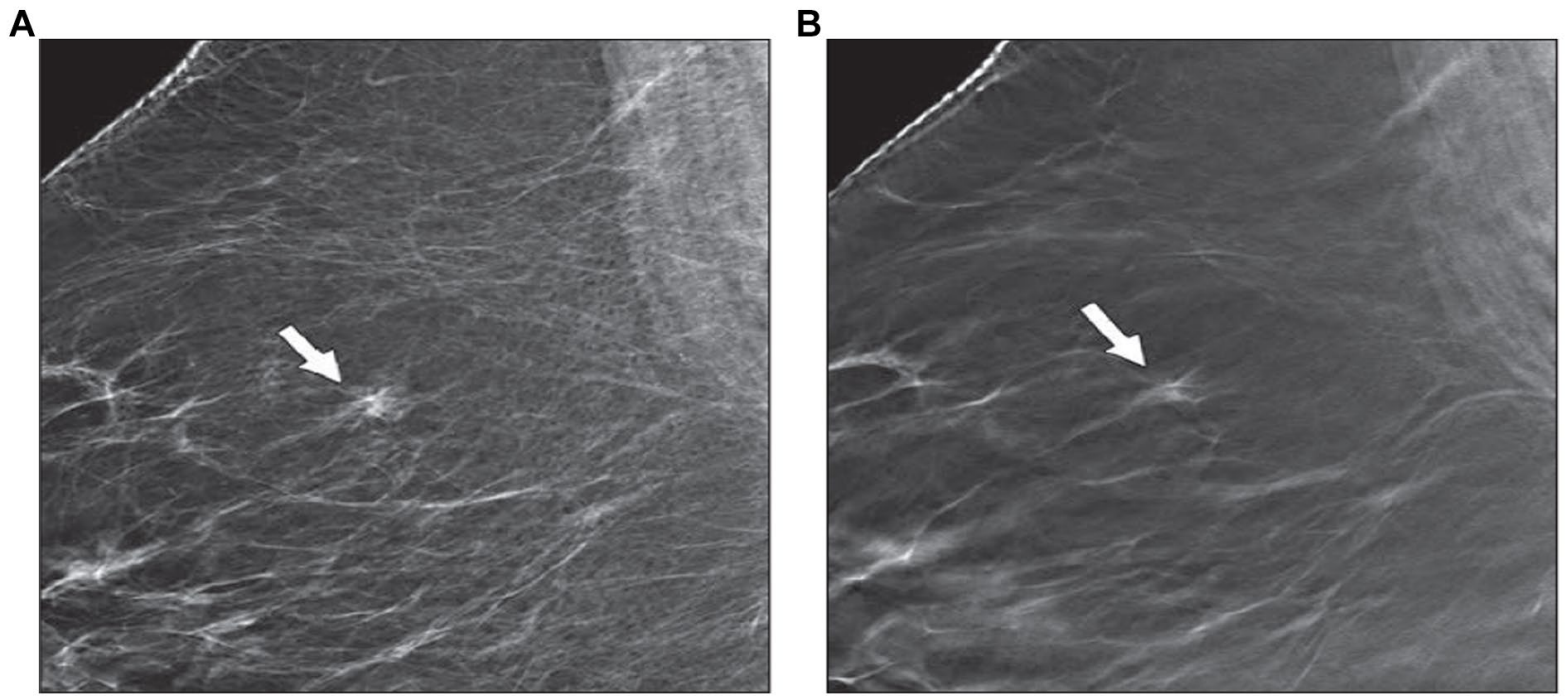
The ability of DBT to resolve asymmetry. There is a symmetry that is seen in the right upper breast in the MLO view of 2D mammography **(A)**. **(B)** DBT image detects crossing cooper ligaments and fibro-glandular tissue (arrow) with no associated mass or speculation (12).

To alleviate the limitations of 2D mammography, digital breast tomosynthesis (DBT) was approved by the Food and Drug Administration (FDA) in 2011 (12). It is an advanced imaging technique that allows the acquisition of multiple low-dose projection images of the breast over a fixed angular range (13). It has been stated that breast cancer screening with DBT plus 2D mammography has the benefit of increasing CDR ranging from 1.2 to 4.6 per 1,000 mammographic examinations when compared with 2D mammography alone (14, 15). The increase in CDR was noticeable in both dense and fatty breasts when using DBT compared with 2D mammography alone due to enhancing the conspicuity of the lesions across all breast densities and cancers unmasking (Figure 1) (17, 18). In addition, another study performed by Gillbert and his colleagues found that the sensitivity, specificity and AUC were significantly higher with the addition of DBT (89, 69% and 0.89, respectively) than with DM (87, 58%, and 0.84, respectively) (19). It has been reported that DBT has been associated with a reduction in recall rates compared with 2D mammography due to the superiority of DBT in resolving asymmetries/ focal asymmetries (Figure 2) (20). Moreover, a previous study compared two groups, one screened with DBT, and the other control group screened with 2D mammography showed lower interval cancer detection after screening with DBT (21). The improvement in lesion conspicuity and characterization of margins offered by DBT allowed for cancer detection at the screening stage and assisted in the diagnostic evaluation (22). It has been shown that the need for additional views was reduced when utilizing DBT, particularly for non-calcified findings. However, magnification views are still required for assessing microcalcifications (23, 24). In addition, a previous retrospective study compared DBT with 2D mammography showed a lower screening recall rate for asymmetries with DBT than 2D mammography (13.3 vs. 32.2%, respectively) and focal asymmetries (18.2 vs. 32.2%). This advantage will contribute positively to reducing the false positive recall rate (25).

Breast ultrasound is adopted as an adjunct screening tool for 2D mammography, particularly for women with dense breasts, due to its superiority in detecting cancers in dense breast tissue, lack of ionizing radiation, and its availability (26, 27). On the other hand, breast ultrasound is limited due to time-consuming, high rate of false positive findings, negative biopsy findings, and operator dependence (28, 29). In addition, due to limited lesion visibility in 2D mammography, many additional examinations are often required in assessing mammographic abnormality, like extra-views (mainly magnification and spot compression views) and biopsies (30). Despite the known benefits of these examinations in increasing diagnostic accuracy, lesion visibility, and lesion characterization, additional risks are involved, particularly in older women, as the benefits of screening mammography in this age group are still controversial (31). These risks include psychological stress, extra radiation dose, and cost (30). In addition, it has been shown that 50% of U/S cases and 21% of biopsies used as adjuncts to 2D mammography were unnecessary (32).

The elderly population can be defined as people who are aged 65 years and over (33). The main reason for choosing older women aged 65 years and older in our study is the increasing incidence of breast cancer with age, so older women have a higher chance of developing breast cancer detected at screening mammography. In addition, 41% of all incident breast cancers and 57% of all breast cancer deaths are among women ages 65 and older (34). Another significant consideration is breast density, which alters during a woman’s life. It has been shown that DBT is particularly beneficial in dense breasts. However, older women have less dense tissue (35). In addition, the number of older women and their life expectancy is increasing (34). So, we must identify the added screening and diagnostic performance of newer imaging techniques, such as DBT, over variable breast density in this target group.

To examine the benefits of DBT in older women (≥65 years) and its ability to reduce unnecessary supplemental examinations, multiple studies in the literature from different research groups discussed the performance of DBT in older women by comparing it to 2D mammography and ultrasound over variable breast densities. The data from the existing literature about using DBT as adjunct screening for dense breasts are diverse (36). Some previous studies indicate the higher diagnostic performance of DBT in detecting lesions in dense breasts (36–38). On the other hand, it was shown that the addition of DBT to 2D mammography was not associated with improvement in the cancer detection rate, particularly for highly dense and almost entirely fatty breasts (39–41). Extremely few scientific studies have been carried out to examine screening outcomes of using DBT compared to 2D mammography, particularly for older women. A recent retrospective study compared the performance indicators of 2D mammography and DBT and found that among women aged 65 years and more, DBT had higher positive predictive value (14.5% vs. 11.9%, *p* = 0.03), higher specificity (95.1% vs. 94.8%, *p <* 0.001), lower abnormal interpretation rate (5.7% vs. 5.8%, *p* < 0.001) and comparable cancer detection rate compared to 2D mammography (42). A second study confirmed the recall rate reduction for women aged 50 to 75 years after comparing (DBT plus 2D mammography) to 2D alone (43).

Prior ultrasound research confirmed that U/S could detect 27% more malignant lesions when used as a supplementary imaging test for 2D mammography (44), while another one shows a similar cancer detection rate compared to 2D mammography (27). Although the usage of DBT and U/S as supplementary imaging tools for 2D mammography is prevalent, only a few previous studies have compared the diagnostic performance of DBT versus U/S, particularly for older women with variable breast densities (45–47). According to the findings of these published studies, there is a variation in the diagnostic performance of both DBT and U/S as adjunct imaging modalities for 2D mammography for older women with variable breast densities. The variation comes from methodology, sample size, and study population. Another study reported that U/S detects more breast cancers than DBT; however, it yields more false positive findings. This study examines mammographically negative cases and includes a small sample size over the age of 65 years (48). Kim et al. found that using DBT performs similarly to U/S for cancer detection, except for highly dense tissue; however, he does include women aged 65 years and older (49).

To the best of our knowledge, only one recent study examined the ability of DBT to reduce subsequent examinations in older women. However, this study did not depend on Breast imaging reporting and data system (BI-RADS) for classifying breast density; instead, it depends on age as a strong association with breast cancer (50). Therefore, this study aims to identify the diagnostic performance of DBT for breast cancer and calcification detection in older patients (aged 65 years and older) across dense and non-dense breasts and to compare the diagnostic performance of both DBT, 2D mammography and U/S for cancer detection and 2D mammogram with DBT for calcification detection. It also aims to describe the number of additional mammographic views, ultrasound, and recommended biopsy that is needed after using DBT and make a prediction of the ability of DBT to reduce it.

## 2 Methodology

### 2.1 Ethical approval

This study has been approved by the Institutional Review Board at Jordan University of Science and Technology. Radiology reports from the mammography department at King Abdullah University Hospital were obtained for analysis at the time of the study. All patients’ and radiologists’ details were de-identified during and after data collection.

### 2.2 Patient selection

Within the mammography unit at King Abdullah University Hospital in Jordan, the screening and diagnostic program for women includes a clinical breast examination, 2D mammography, mammography spot views, mammography magnification views, DBT, ultrasound, and a percutaneous biopsy when required. All women aged 65 years and older who underwent 2D mammography and DBT examinations were included in this study; the selection of the patients was random regardless of patient background. For these patients, spot compression views, magnification views, ultrasound, and recommended biopsy were performed whenever required. After applying the inclusion criteria, 224 women were included in the study. The data was collected between the period of January 2023 to April 2023. Those selected patients were all patients who performed both 2D mammography and DBT from the implementation of DBT in King Abdullah University Hospital which was in January 2021 to April 2023. Imaging procedures such as DBT and 2D mammography were performed for all patients, and additional examinations like U/S or additional mammographic views were performed on the same appointment whenever required.

### 2.3 Study design

We retrospectively reviewed the radiologist’s reports for all included women. In the breast screening and diagnostic mammogram at King Abdullah University Hospital, cases were interpreted by one experienced radiologist trained in mammography image interpretation and devoted to breast imaging. The classification of lesions in the Jordan breast screening and diagnostic program is based on the (BIRADS) system for breast imaging lesion classification. This classification system is based on a simple 0–6 grading scale: 0 = “incomplete examination,” 1 = “normal finding,” 2 = “benign,” 3 = “probably benign (needs to follow up),” 4 = “suspicious (advised biopsy),” 5 = “high suggestive of malignancy,” 6 = “confirmed by biopsy (malignant)” (51). Two mammographic views were obtained for the examined breast: craniocaudal (CC) and mediolateral oblique (MLO). A single MLO view was obtained for the DBT image. Further mammography spot and magnification views were acquired if deemed necessary. Breast density was categorized according to (BI-RADS) system:

- BI-RADS A: “The breasts are almost entirely fatty.”
- BI-RADS B: “There are scattered areas of fibro-glandular tissue.”
- BI-RADS C: “The breasts are heterogeneously dense.”
- BI-RADS D: “The breasts are extremely dense.”

DBT and ultrasound examinations were performed for selected patients before they were referred for biopsies. DBT examination was performed using Selenia 3Dimensions, Hologic. Multiple radiologic features were used to describe lesions identified in 2D mammography and DBT, like calcifications, focal asymmetry, architectural distortion, discrete mass, and multiple masses. Breast ultrasound with real-time B-mode was performed using LOG IQI Ultrasound.

System (HELX Evolution with Touch Control, TOSHIPA Medical Solutions), equipped with a linear array transducer (7.5 MHz (frequency)). For the characterization of breast lesions, color Doppler was also used. The sonographic features of ultrasound lesions include indeterminate, cystic, solid, and solid mass (probably benign) and solid (probably malignant). Lesions detected on DBT and ultrasound were also rated using the BIRADS breast imaging lesion classification scale. One radiologist interpreted both ultrasound and DBT depending on the digital mammographic findings.

### 2.4 Statistical analysis

Data from patient reports (which represents the findings of each imaging technique) combined with the overall conclusion (represents BIRADS classification of the lesion) was transferred manually to Excel sheet version 2007 and then to (SPSS) version 24 for statistical analysis. Before analysis, all variables were checked for accuracy of data entry and missing values. For analysis, we assigned the number (1) for the positive finding (lesion was present) in each examining procedure (2D, DBT, and U/S) compared to the final reference between image findings, and we assigned the number (0) for the negative finding (lesion was absent). For calcification detection between 2D mammography and DBT, we compare the presence or absence of calcification. Similarly, we assigned the number (1) for the presence of calcification and the number (0) for the absence of calcification depending on the findings of both imaging techniques, and we exclude U/S from this comparison due to its known limitation for calcification detection (52). For breast density, cases categorized as BI-RADS A and B were considered non-dense breasts, and those classified as BI-RADS C and D were considered dense breasts. Finally, we displayed the characterization of lesions detected according to the (BIRADS) scale.

Descriptive statistics summarized frequencies and percentages for demographics and categorical variables. The diagnostic performance of 2D mammography, DBT, and U/S was calculated in terms of sensitivity, specificity, positive predictive value (PPV) and negative predictive value (NPV), and area under the curve (AUC) of the receiver operator characteristics (ROC) curve across dense and non-dense breasts. Sensitivity, specificity, positive predictive value (PPV), and negative predictive value (NPV) were calculated using cross-tabulation. A generalized linear model calculated confidence intervals for sensitivity, specificity, PPV, and NPV. Discrimination values were calculated using the area under the curve (AUC) of the receiver operator characteristics (ROC) curve. The predictability of reducing additional measures (ultrasound necessity, magnification view necessity, spot compression view necessity, and biopsy necessity) after DBT was assessed using binary logistic regression, which was run four times. A value of *p* ≤ 0.05 was considered statistically significant.

## 3 Results

### 3.1 Characteristics of examined patients

Table 1 summarizes the demographic and clinical characteristics of 224 women (mean age 71.1 SD 4.64 years) included in this study according to radiology reports that were analyzed retrospectively. All these women underwent digital mammography and digital breast tomosynthesis examinations. Spot compression views, magnification views, ultrasound, and recommended biopsy were performed whenever required. According to the table, more than half of women, 54% (*n* = 121), were aged between 65 and 70 years, while only 1.3% (*n* = 3) of women were over 82 years. 62.1% (*n* = 139) of the sample had non-dense breasts, which are divided into scattered areas of fibro-glandular tissue 40.2% (*n* = 90) and almost entirely fatty breasts 21.9% (*n* = 49). 38% (*n* = 85) of sample had dense breasts; 36.2% (*n* = 81) were heterogeneously dense, and 1.8% (*n* = 4) were extremely dense. Regarding the examined breast side, 75.4% (*n* = 169) of the sample had both sides examined. In comparison, only 13.8% (*n* = 31) had the right side, and 10.7% (*n* = 24) had the left side examined (those patients were symptomatic on one side or may have had a follow-up examination on one breast after mastectomy). Regarding BI-RADS classification of lesions that have been detected, 65.6% (*n* = 147) of lesions were benign, 10.7% (*n* = 24) of lesions were probably benign (needs to follow up), 15.2% (*n* = 34) of lesions were suspicious (advised biopsy), 5.4% (*n* = 12) of lesions were high suggestive of malignancy, and 3% (*n* = 7) of lesions were confirmed by biopsy as (malignant).

**Table 1:** Patient age and imaging characteristics.

Characteristics		*N*	%
Age in years	65–70	121	54
	71–76	69	30.8
	77–82	31	13.8
	>82	3	1.3
Breast density	Almost entirely fatty	49	21.9
	Scattered areas of fibro-glandular tissue	90	40.2
	Heterogeneously dense	81	36.2
	Extremely dense	4	1.8
The examined breast side	Rt Side	31	13.8
	LT side	24	10.7
	Rt and Lt sides	169	75.4
The BI-RAD classification of lesions	Incomplete examination	0	0
	Normal finding	0	0
	Benign	147	65.6
	Probably benign (needs follow-up)	24	10.7
	Suspicious (advised biopsy)	34	15.2
	High suggestive of malignancy	12	5.4
	Confirmed by biopsy (malignant)	7	3

### 3.2 Performance measures of DBT across breast density

Table 2 shows performance measures of DBT for cases with different breast densities. According to the table, the sensitivity of DBT in dense breasts was 61.9% (CI: 0.847–0.998) compared with 49.3% (CI: 0.877–0.998) for non-dense breasts *p* < 0.001. The specificity for lesion detection in dense and non-dense breasts of DBT was 100% (CI: 0.607–0.831) and (CI: 0.587–0.763), respectively, *p* < 0.001. The PPV for lesion detection in dense and non-dense breasts of DBT was 100% (CI: 0.468–0.756) and (CI: 0.375–0.611), respectively *p* < 0.001. The NPV for dense breasts was 72.9% (CI: 0.904–0.999) compared with 67.9% (CI: 0.941–0.999) in non-dense breasts *p* < 0.001. Regarding the DBT discrimination ability between positive and negative lesions, in dense breasts was 0.425 (95% CI: 0.203–0.647), compared with 0.670 in non-dense breasts (95% CI: 0.473–0.868). For dense breasts, the sensitivity of DBT for calcification detection was 100% (CI: 0.947–0.999), compared with 99.2% (0.964–1) in non-dense breasts *p* < 0.001. The specificity in dense and non-dense breasts was 100% (CI: 0.372–0.987) and (CI: 0.788–0.997), respectively, *p* < 0.001. Likewise, the PPV in dense and non-dense breasts was 100% (CI: 0.947–0.999) and (CI: 0.964–1), respectively, *p* < 0.001. The NPV in dense breasts of DBT was 100% (CI: 0.372–0.987), which was higher than non-dense breasts of 94.7% (CI: 0.788–0.997) *p* < 0.001. DBT ability of discrimination between positive and negative calcification (AUC) in dense breasts was 0.682 (95% CI: 0.480–0.884), while in non-dense breasts was 0.711 (95% CI, 0.609–0.814).

**Table 2:** Performance measures of DBT across breast density.

Diagnostic performance % (95% CI)	Lesion detection		Calcification detection	
	Dense	Non-dense	Dense	Non-dense
Sensitivity	61.9 (0.847–0.998)	49.3 (0.877–0.998)	100 (0.947–0.999)	99.2 (0.964–0.1)
*p*	<0.001	<0.001	<0.001	<0.001
Specificity	100 (0.607–0.831)	100 (0.587–0.763)	100 (0.372–0.987)	100 (0.788–0.997)
*p*	<0.001	<0.001	<0.001	<0.001
PPV	100 (0.468–0.756)	100 (0.375–0.611)	100 (0.947–0.999)	100 (0.964–1)
*p*	<0.001	<0.001	<0.001	<0.001
NPV	72.9 (0.904–0.999)	67.9 (0.941–0.999)	100 (0.372–0.987)	94.7 (0.788–0.997)
*p*	<0.001	<0.001	<0.001	<0.001
ROC AUC	0.425 (0.203–0.647)	0.670 (0.473–0.868)	0.682 (0.480–884)	0.711 (0.609–0.814)
*p*	0.542	0.089	0.053	<0.001

### 3.3 The diagnostic performance of 2D mammography, U/S, and DBT for lesion detection for all cases

According to Table 3, the sensitivity of 2D mammography was 91.7% (CI: 0.957–0.999) compared to 83.7% (CI: 0.929–0.999) for DBT, while ultrasound was 60.6% (CI: 0.512–0.694), *p* < 0.001. The specificity in 2D mammography, U/S, and DBT was 100% (CI: 0.963–1); (CI: 0.963–1); and (CI: 0.624–1) respectively *p* < 0.001. Similarly, the PPV of 2D mammography, U/S, and DBT was 100% (CI: 0.957–0.999); (CI: 0.963–0.999); and (CI: 0.448–0.633) respectively *p* < 0.001. The NPV of DBT was 40% (CI: 0.963–1), which was lower than that of ultrasound 72.8% (CI: 0.655–0.793), and 2D mammogram 92.7% (CI: 0.873–964). Regarding discrimination ability between positive and negative lesions (AUC) among imaging procedures, 2D mammography is the highest one, 0.725 (95% CI: 0.610–0.840), followed by U/S 0.664 (95% CI: 0.515–0.813) and the least is DBT 0.573 (95% CI: 0.417–0.730).

**Table 3:** The diagnostic performance of 2D mammography, DBT, and ultrasound for lesion detection for all cases.

Diagnostic performance% (95% CI)	Modality		
	2D mammography	Ultrasound	DBT
Sensitivity	91.7 (0.957–0.999)	60.6 (0.512–0.694)	83.7 (0.929–0.999)
*p*	<0.001	<0.001	<0.001
Specificity	100 (0.963–1)	100 (0.963–1)	100 (0.624–1)
*p*	<0.001	<0.001	<0.001
PPV	100 (0.957–0.999)	100 (0.963–0.999)	100 (0.448–0.633)
*p*	<0.001	<0.001	<0.001
NPV	92.7 (0.873–964)	72.8 (0.655–0.793)	40 (0.963–1)
*p*	<0.001	<0.001	<0.001
ROC AUC	0.725 (0.610–0.840)	0.664 (0.515–813)	0.573 (0.417–0.730)
*p*	0.004	0.034	0.344

### 3.4 The diagnostic performance of 2D mammography, U/S, and DBT for lesion detection across breast density

According to Table 4, the sensitivity of DBT for lesion detection in dense breasts is 61.9% (CI: 0.847–0.998), which is significantly lower than that of ultrasound 76.2% (CI: 0.873–0.998), and 2D mammography 92.9% (CI: 0.895–0.999) *p* < 0.001. In addition, sensitivity for lesion detection of DBT in non-dense breasts was 49.3% (0.877–0.998), which was slightly lower than that of ultrasound 50.7% (0.880–0.998) and significantly lower than that of 2D mammography 91% (0.931–0.999) *p* < 0.001. The specificity for lesion detection in dense breasts under 2D mammography, U/S, and DBT was the same 100% with the following confidence interval values (CI: 0.840–0.983); (CI: 0.693–0.901); and (CI: 0.607–0.831) respectively *p* < 0.001. Similarly, the specificity for lesion detection in non-dense breasts under 2D mammography, U/S, and DBT was the same at 100% with the following confidence interval values (CI: 0.850–0.969); (CI: 0.593–0.769); and (CI: 0.587–0.763) respectively *p* < 0.001. Likewise, the PPVs for lesion detection in dense breasts under 2D mammography, U/S, and DBT were 100% (CI: 0.825–0.982); (CI: 0.620–0.873); and (CI: 0.468–0.756) respectively *p* < 0.001.

**Table 4:** The diagnostic performance of 2D mammography, DBT, and U/S for lesion detection according to breast density.

Diagnostic performance % (95% CI)	Modality					
	2D mammography		Ultrasound (U/S)		DBT	
	Dense	Non-dense	Dense	Non-dense	Dense	Non-dense
Sensitivity	92.9 (0.895–0.999)	91 (0.931–0.999)	76.2 (0.873–0.998)	50.7 (0.880–0.998)	61.9 (0.847–0.998)	49.3 (0.877–0.998)
*p*	<0.001	<0.001	<0.001	<0.001	<0.001	<0.001
Specificity	100 (0.840–0.983)	100 (0.850–0.969)	100 (0.693–0.901)	100 (0.593–0.769)	100 (0.607–0.831)	100 (0.587–0.763)
*p*	<0.001	<0.001	<0.001	<0.001	<0.001	<0.001
PPV	100 (0.825–0.982)	100 (0.827–0.963)	100 (0.620–0.873)	100 (0.389–0.625)	100 (0.468–0.756)	100 (0.375–0.611)
*p*	<0.001	<0.001	<0.001	<0.001	<0.001	<0.001
NPV	93.5 (0.904–0.999)	92.3 (0.941–0.999)	81.1 (0.904–0.999)	68.6 (0.941–0.999)	72.9 (0.904–0.999)	67.9 (0.941–0.999)
*p*	<0.001	<0.001	<0.001	<0.001	<0.001	<0.001
ROC AUC	0.791 (0.671–0.911)	0.681 (0.512–0.850)	0.746 (0.557–0.934)	0.607 (0.404–0.810)	0.425 (0.203–0.647)	0.670 (0.473–0.868)
*p*	0.018	0.070	0.046	0.285	0.542	0.089

Additionally, the PPVs in non-dense breasts for 2D mammography, U/S, and DBT were 100% (CI: 0.827–0.963); (CI: 0.389–0.625); and (CI: 0.375–0.611) respectively *p* < 0.001. The NPV of DBT in dense breasts was 72.9% (CI: 0.904–0.999), which is significantly lower than both U/S 81.1% (CI: 0.904–0.999), and 2D mammogram 93.5% (CI: 0.895–999) *p* < 0.001. Moreover, The NPV of DBT in non-dense breasts is 67.9% (CI: 0.941–0.999), which is slightly lower than that of U/S 68.6% (CI: 0.941–0.999) and significantly lower than that of 2D mammography 92.3% (CI: 0.941–999) *p* < 0.001. Regarding the discrimination ability of imaging procedures between positive and negative lesions in dense breasts, the AUC value for 2D mammography was 0.791 (95% CI: 0.671–0.911), and U/S 0.746 (95% CI: 0.557–0.934), both were better than DBT 0.425 (95% CI: 0.203–0.647). Similarly, the AUC value for 2D mammography in non-dense breasts was 0.681 (95% CI: 0.512–0.850), better than DBT 0.670 (95% CI: 0.473–0.868). However, DBT here is better than U/S 0.607 (95% CI: 0.404–0.810).

### 3.5 The diagnostic performance of DBT versus 2D mammography for calcification detection according to breast density

According to Table 5, the sensitivity of DBT for detecting calcifications in dense breasts was 100% (CI: 0.947–0.999), which was significantly higher than that of 2D mammogram 91.4% (CI: 0.943–0.999) *p* < 0.001. In addition, the sensitivity of DBT for calcification detection in non-dense breasts was 99.2 (0.964–1), which was significantly higher than that of 2D mammography 78.5 (0.941–0.998) *p* < 0.001. The specificity was the same in both 2D mammography and DBT in dense breasts 100% (CI: 0.130–0.654) and (CI: 0.372–0.987), respectively, *p* < 0.001. Similarly, the specificity in non-dense breasts in both 2D mammography and DBT was the same at 100% (CI: 0.272–0.556) and (CI: 0.788–0.997), respectively, *p* < 0.001. Likewise, the PPVs of both 2D mammography and DBT in dense breasts were the same at 100% (CI: 0.840–0.962) and (CI: 0.947–0.999), respectively, *p* < 0.001. Additionally, the PPVs of both 2D mammography and DBT in non-dense breasts were the same at 100% (CI: 0.356–0.625) and (CI: 0.964–1), respectively, *p* < 0.001. The NPV of DBT in dense breasts was 100% (CI: 0.372–0.987), which was significantly higher than that of 2D mammography at 36.4% (CI: 0.372–987) *p* < 0.001. Moreover, The NPV of DBT in non-dense breasts was 94.7% (CI: 0.788–0.997), which is also significantly higher than that of 2D mammography at 40.9% (CI: 0.788–997) *p* < 0.001. Regarding the discrimination ability of 2D mammography and DBT between positive and negative calcifications in dense breasts, the AUC value of 2D mammography was 1 (95% CI: 1–1), which was better than DBT 0.682 (95% CI: 0.480–0.884). Likewise, in non-dense breasts, 2D mammography 0.989; (95% CI: 0.964–1) was better than DBT 0.711 (95% CI, 0.609–0.814).

**Table 5:** Diagnostic performance of 2D mammography and DBT for calcification detection across breast density.

Diagnostic performance % (95% CI)	Modality			
	2D mammography		DBT	
	Dense	Non-dense	Dense	Non-dense
Sensitivity	91.4 (0.943–0.999)	78.5 (0.941–0.998)	100 (0.947–0.999)	99.2 (0.964–0.1)
*p*	<0.001	<0.001	<0.001	<0.001
Specificity	100 (0.130–0.654)	100 (0.272–0.556)	100 (0.372–0.987)	100 (0.788–0.997)
*p*	<0.001	<0.001	<0.001	<0.001
PPV	100 (0.840–0.962)	100 (0.356–0.625)	100 (0.947–0.999)	100 (0.964–1)
*p*	<0.001	<0.001	<0.001	<0.001
NPV	36.4 (0.372–0.987)	40.9 (0.788–0.997)	100 (0.372–0.987)	94.7 (0.788–0.997)
*p*	<0.001	<0.001	<0.001	<0.001
ROC AUC	1 (1–1)	0.989 (0.964–1)	0.682 (0.480–884)	0.711 (0.609–0.814)
*p*	<0.001	<0.001	0.053	<0.001

### 3.6 The need for supplementary imaging techniques and views after using DBT

According to the results summarized in Table 6, 97.3% (*n* = 218) of cases examined by DBT still require additional ultrasound. On the contrary, only 6.3% (*n* = 14) of cases imaged by DBT required an additional magnification view. Of those cases which required additional magnification view after DBT, 4% (*n* = 9) were cranio-caudal, and 2.2% (*n* = 5) were mediolateral oblique. Moreover, 30.8% (*n* = 69) of cases examined by DBT required additional spot compression views. Of those cases requiring additional spot compression after DBT, 23.2% (*n* = 52) were craniocaudal, and 7.6% (*n* = 17) were mediolateral oblique. Furthermore, biopsy recommendation was 23.7% (*n* = 53) of all cases examined by DBT.

**Table 6:** Additional examinations/ views may or may not be required after DBT use.

Additional measures		*N*	%
The need for additional ultrasound (U/S)	Not required	6	2.7
	Required	218	97.3
The need for an additional magnification view	Not required	210	93.8
	Required	14	6.3
The need for a spot compression view	Not required	155	69.2
	Required	69	30.8
The recommended biopsy cases	Not recommended	171	76.3
	Recommended	53	23.7
The need for an additional magnification view	CC	9	4
	MLO	5	2.2
	No need	210	93.8
The need for a spot compression view	CC	52	23.2
	MLO	17	7.6
	No need	155	69.2

### 3.7 The predictability for the need of additional procedures (U/S, magnification view, spot compression view, and recommended biopsy) based on DBT use

Table 7 shows the logistic regression model, which was run four times between the predictor variable (DBT) and the four additional procedures (U/S, magnification view, spot compression view, and recommended biopsy, respectively). Binary logistic regression was used, and data were examined to meet the assumptions necessary for the Logistic regression analysis. The table shows that the value of p was not significant >0.05 between DBT (predictor variable) and the four respondent variables. The most significant value in this model is the *B* coefficient of the predictor variable, which is DBT used with the four dependent variables with specified *p*-value; according to the table, we can see that the *B* value was not significant for the determined *p* values as all of them were >0.05 so we conclude that DBT was not effective in reducing the additional examinations that we measured.

**Table 7:** Prediction of additional measures based on DBT use.

Predictors	*B*	*SE B*	Wald	*p* - value	Odds ratio Exp (B)	95% CI for Exp (B)	
						Upper	Lower
*Prediction of additional ultrasound requirement based on DBT use*							
Constant	3.491	0.414	70.979	<0.001	32.833		
DBT use	−17.711	8770.825	0	0.998	49202279.480	0	0.215
*Prediction of additional magnification view requirement based on DBT use*							
Constant	−2.682	0.287	87.527	<0.001	0.068		
DBT use	−0.314	1.064	0.087	0.998	0.768	0.731	0.091
*Prediction of additional spot compression view based on DBT use*							
Constant	−0.730	0.150	23.758	<0.001	0.482		
DBT use	−1.061	0.641	2.739	0.098	0.346	0.098	1.216
*Prediction of recommended biopsy requirement based on DBT use*							
Constant	−1.066	0.161	43.957	<0.001	0.344		
DBT use	−1.930	1.037	3.461	0.063	0.145	0.019	1.109

## 4 Discussion

To the best of my knowledge, this is the first study in Jordan that explores and compares the diagnostic performance of DBT, 2D mammography, and U/S among older women and measures the ability of DBT to reduce unnecessary follow-up examinations. The results of this study indicate that DBT was not associated with lower follow-up examinations when compared with 2D mammography but was associated with a higher rate of calcification detection. This agrees with previously published work (50, 53).

The study results demonstrate higher sensitivity and NPV metrics of DBT for dense breasts than non-dense for both lesion and benign calcification detection. However, AUC values were higher in non-dense tissue. These findings were consistent with the previously published work (36–38, 54, 55). In addition, higher similar specificity and PPVs between dense and non-dense tissue could be explained by the radiologist’s experience in DBT image interpretation during the routine reading of images. An interesting finding in this study is that sensitivity for lesion detection in dense tissue was higher than in non-dense breasts; this could be explained by the fact that some of the lesions in dense tissue may be classified as interval cancers or lesions that were missed at previous 2D mammography screening due to its limitation of tissue superimposition (56, 57). Regarding AUC values, the only significant value was reported in calcification detection in non-dense breasts. This means that the discrimination power for positive and negative calcification was more accurate (statistically) in non-dense tissue.

This study reveals that sensitivity, NPV, and AUC values for 2D mammography were higher than DBT (*p* < 0.001). This finding differs from previous studies (58–60). This is because we include focal asymmetry as a sign of non-mass lesion detection, a positive finding in 2D mammography, and the summation artifact that mimics the lesion’s appearance. However, in these cases, this asymmetry had effaced when using DBT due to its advantage of resolving asymmetry, so it is considered a negative finding in DBT (compared with a positive finding in the reference), which will contribute negatively to the performance measures of DBT in lesion detection. In addition, this could be explained by the nature of the lesions that were detected, which were commonly benign lesions that did not show the real ability of DBT to detect lesions.

The higher sensitivity value of 2D mammography compared with U/S was similar to a previous work that stated the increasing sensitivity of 2D mammography with age compared with U/S (61). In addition, the findings of this study demonstrate higher sensitivity of DBT compared with U/S, which is comparable to previous work results (62). Similar higher specificity and PPV percentages between imaging examinations could be explained by radiologist experience in image interpretation and routine reading for 2D mammography, DBT, and U/S images, which was previously stated to be an essential factor that affects the specificity of screening mammograms (63). Also, this finding means that imaging examinations’ diagnostic performance may be enhanced with increasing age. Low NPV for DBT compared with 2D mammography and U/S could be due to the effect of the prevalence rate on reducing the negative predictive value of DBT.

Regarding the diagnostic performance of imaging procedures across breast density, this study reveals that the sensitivity, NPV, and AUC performance metrics in dense breasts were higher for 2D mammography compared with non-dense breasts on both U/S and DBT. The higher diagnostic performance metrics of 2D mammography in dense tissue compared with DBT concord with the findings of previous work (64). On the other hand, these findings contradict the other results stated by previous works (42, 43, 58, 65). The variations in the findings of this study compared with the previously published works could be explained by the appearance of non-mass lesions in 2D mammography, represented by focal asymmetry and architectural distortion, considered a positive lesion in the breast tissue. This factor will positively enhance the sensitivity of 2D mammography compared with DBT. However, this summation of tissue was effaced on DBT. In addition, this could be explained by the nature of the lesions that were detected, which were commonly benign lesions that did not show the real ability of DBT to detect lesions. Moreover, the higher diagnostic performance metrics of 2D mammography in dense tissue compared with U/S were comparable with prior work (44).

The higher diagnostic performance of U/S in dense breasts compared with DBT was consistent with prior studies (48, 49). However, this finding differed from those published in prior studies, which stated that DBT had higher diagnostic performance than U/S in dense breasts (62, 66). Our findings differ from previous studies due to the following reasons. First, some lesions may be obscured by dense tissue even when using DBT, as well as DBT only provides morphological information about lesions (49). Second, the usage of Doppler sonography had the advantage of lesion characterization that provides differentiation between benign and malignant lesions. Similar PPV and specificity metrics between imaging procedures may reflect radiologists’ experience and skill in image interpretation during routine reading in screening and diagnostic settings.

This study reveals that sensitivity and NPV metrics for benign calcification detection were higher in DBT compared with 2D mammography for dense and non-dense tissue (*p* < 0.001). This finding was expected due to DBT’s ability to take multiple projections from different angles and thus increase the detectability of calcification detection, which was comparable with the prior studies (53). Similar higher PPV and specificity metrics were stated in this comparison due to the same reason mentioned previously. AUC values were higher in 2D mammography than DBT values for both dense and non-dense tissues with statistical significance, reflecting more accuracy in the discrimination power among 2D mammography.

This study’s results demonstrate that many U/S examinations are still required after using DBT. This finding indicates the value of U/S as an adjunct modality to 2D mammography to detect occult cancers in dense breasts that may be missed in both 2D mammography and DBT, as well as lesion characterization value due to the advantage of using Doppler sonography even when using DBT (67). Similarly, all recommended biopsy cases, which were usually recommended for biopsy (suspicious, highly suggestive of malignancy, and confirmed by biopsy (malignant) lesions), BIRADS 4, 5 and 6, respectively, are still recommended after using DBT. Per our expectation, despite the advantages of DBT for providing morphological information and resolving asymmetries, we still need a biopsy to confirm these lesions as benign or malignant. On the contrary, a few additional magnification and spot compression views were required after using DBT. Although we do not measure the statistical significance of this descriptive finding, this finding was consistent with prior studies (68). According to this result, we could suggest that DBT may be considered as a substitute for additional mammographic views.

The results of logistic regression models that predict the effect of the predictor variable (DBT) on the four dependent variables (U/S, magnification view, spot compression view, and recommended biopsy) indicate no statistical significance as value of *p* > 0.05 in the four models. This finding concorded a prior study (50). This finding suggests that DBT was not associated with reducing subsequent imaging among older women.

The main limitation of this study was that we did not include more than one hospital. However, DBT is only implemented in a small number of hospitals in Jordan that selectively perform DBT examinations, not for all patients. Also, we suffered from a small sample size for all cases and between subgroups. On the other hand, DBT is newly implemented in King Abdullah University Hospital, and we included all older patients that DBT has examined since its implementation. Further studies with large samples and confirmed biopsy results are recommended to strongly generalize the effectiveness of using DBT as a screening and diagnostic tool in older women.

The main limitation of this study was that we did not include more than one hospital. However, DBT is only implemented in a small number of hospitals in Jordan that selectively perform DBT examinations, not for all patients. Also, we suffered from a small sample size for all cases and between subgroups. On the other hand, DBT is newly implemented in King Abdullah University Hospital, and we included all older patients that DBT has examined since its implementation. The accuracy results were not assessed against pathology results because it falls outside the study’s scope. Further studies with large samples and confirmed biopsy results are recommended to strongly generalize the effectiveness of using DBT as a screening and diagnostic tool in older women.

## 5 Conclusion

This study showed that sensitivity and NPV metrics of DBT were higher in dense tissue than in non-dense tissue for both lesion and calcification detection. Similar high specificity and PPV between dense and non-dense tissue indicated the diagnostic improvement of DBT over both tissues. This finding highlights the diagnostic value of DBT in detecting lesions and calcifications in dense tissue that may be missed during another imaging procedure. During comparison between imaging examinations for lesion detection in all cases, DBT showed a lower sensitivity metric than 2D mammography but higher than U/S. This finding indicated that older women may not benefit from using DBT as an adjunct modality to 2D mammography for lesion detection. The performance metrics of DBT for lesion detection across breast density were lower than both 2D mammography and U/S, indicating that we still need 2D mammography and U/S for detecting lesions that may be difficult to detect even when using DBT. DBT was superior to 2D mammography in the detectability of calcifications. Also, DBT was not associated with reducing additional follow-up examinations among older women.

## Data availability statement

The raw data supporting the conclusions of this article will be made available by the authors, without undue reservation.

## Author contributions

MG: Writing – original draft. AA: Writing – original draft. EE-O: Writing – original draft. RK: Writing – original draft. LR: Writing – original draft. ME-H: Writing – original draft. MA-J: Writing – original draft. AH: Writing – original draft. MA: Writing – original draft. LA: Writing – original draft.
